# Unraveling the associations of osteoprotegerin gene with production traits in a paternal broiler line

**DOI:** 10.1186/2193-1801-3-682

**Published:** 2014-11-20

**Authors:** Marcelo B Fornari, Ricardo Zanella, Adriana MG Ibelli, Lana T Fernandes, Maurício E Cantão, Vanete Thomaz-Soccol, Mônica C Ledur, Jane O Peixoto

**Affiliations:** Departamento de Engenharia de Bioprocessos e Biotecnologia, Universidade Federal do Paraná, Curitiba, PR Brazil; Laboratório de Genética e Melhoramento Animal, Embrapa Suínos e Aves, BR 153, km 110, Distrito de Tamanduá, Caixa Postal 21, 89700-000 Concórdia, SC Brazil

**Keywords:** Bone metabolism, Bone resistance, Chicken, Fat deposition, *TNFRSF11B*

## Abstract

Improvements on growth and carcass traits in the poultry industry have been achieved by intense selection for heavier chickens at early ages. This faster growth has caused serious problems due to insufficient skeletal structure development needed to support the musculature of modern broilers. The osteoprotegerin gene (*OPG*), located on GGA2, is an important regulator of bone metabolism and reabsorption, being suggestive as a possible functional candidate gene associated with bone integrity in chickens. This study reports associations of a single nucleotide polymorphism (SNP) in the *OPG* gene with production traits in a parental broiler line. Different phenotypic groups were evaluated: performance, carcass and skeletal traits. SNPs were identified within the *OPG* gene and the most informative SNP g.9144C > G was chosen for association analyses. Chickens (n = 1230) were genotyped using PCR-RFLP. The association was carried out with QxPaK v4.0 software using a mixed model including sex, hatch and SNP as fixed effects, and the infinitesimal and residual as random effects. The *OPG* SNP was associated with important traits as body weight at 21 days, weights of tibia and drumstick skin, leg muscle yield, and tibia breaking strength (P < 0.05). Associations were explained by the additive effect of the SNP and the additive effect within sex. This SNP could be considered a potential marker to improve bone resistance in chickens; however, caution should be taken because of its negative effect in other important traits evaluated in this study. Furthermore, these findings suggest a possible involvement of the *OPG* gene in fat deposition in poultry.

**Electronic supplementary material:**

The online version of this article (doi:10.1186/2193-1801-3-682) contains supplementary material, which is available to authorized users.

## Introduction

Considerable advances in poultry breeding programs have been observed in the last decades leading to a more efficient chicken production (Havenstein et al. 2003). This improvement sped up the genetic gain of important production traits, such as growth rate, carcass yield and feed efficiency (Li et al. 2003). The intense selection for rapid growth or high egg production rate, together with the intensification of the production system in the poultry industry, have negatively impacted the quality of the skeleton structure in both laying hens and meat-type chickens. These have resulted in an increased number of chickens with bone-related problems (Zhou et al. 2007; Zhang et al. 2010). Disorders affecting the skeletal structure are multifactorial (Cook, 2000). Nutrition, genetics, pathogens, mycotoxins and management practices are some of the factors that directly affect the normal growth and development of bone tissues (Cook, 2000).

Since 1930, numerous causes of deformities have been identified in chicken bone. Problems associated with the locomotor system are generating huge economic losses and creating welfare concerns. Most of the measurable economic losses are caused by an increase in mortality, carcass condemnation, and a reduction in carcass yield (Cook, 2000; Rath et al. 2000; Powell and Bittar, 2008). The non-measurable losses are low performance due the restriction of food and water, and an increase in animal welfare issues (Zhang et al. 2010).

Bone is a dynamic tissue influenced by many physiological, nutritional and physical factors (Rath et al. 2000). Genetics is an important factor that plays a considerable role in the development of the skeletal system (Cook, 2000; Zhou et al. 2007). Actually, genetic companies have made efforts to reduce the incidence of these skeleton abnormalities. Several ways to diagnose bone integrity problems are being developed and applied to improve the quality of the skeletal system. However, the genetic progress achieved has been insufficient to eliminate those problems. Recent advances in genomics have made it possible to investigate new ways to improve the skeletal structure in chickens without affecting animal performance (Ray et al. 2006; Zhou et al. 2007).

Some genes are known to be involved with the molecular mechanisms associated with formation, development and maintenance of bone tissue, such as the *Bone Morphogenetic Proteins* (BMPs), *osteopontin*, *osteoprotegerin*, and the *Vitamin D receptor* (VDR) genes. Although there is a potential large number of functional candidate genes involved with bone metabolism, there are few association studies with bone integrity traits in chickens (Bennet et al. 2006). Bennet et al. ( 2006) investigated four biological candidate genes (*vitamin D receptor*, *VDR*; *insulin*, *INS*; *insulin*-*like growth factor 1*, *IGF1*; *and osteopontin*, *SPP1*) in a layer × broiler F2 chicken resource population. Significant associations (P < 0.0125) were found between *VDR* and bone mineral content of the humerus at 35 weeks of age.

Besides candidate gene studies, some quantitative trait loci (QTLs) have been mapped for bone integrity-related traits in chickens. Schreiweis et al. ( 2005) reported 19 QTL influencing bone size in an F2 broiler-layer cross. One significant QTL on chromosome 1 was mapped for bone index and for its component traits: tibial and humeral breaking strength in an F2 chicken population produced from lines of hens that had been divergently selected for bone index (Dunn et al. 2007). Other important QTL for 12 bone integrity traits located on 14 chromosomes were also mapped in another F2 chicken population (Zhou et al. 2007). From those, QTLs for tibia mineral density (Zhou et al. 2007), tibia mineral content, tibia width (Schreiweis et al. 2005) and tibia length (Zhou et al. 2007) were mapped to a region of GGA2 where an interesting candidate gene for bone integrity is located: the osteoprotegerin gene (*OPG*).

The *OPG* belongs to the TNF receptor family and was simultaneously discovered in 1997 by two groups (Simonet et al. 1997; Tsuda et al. 1997). *OPG* is also known as tumor necrosis factor receptor superfamily member 11B (*TNFRSF11B*) and it is a key factor to inhibit osteoclast activation and differentiation by acting as a decoy receptor of the *Receptor Activator of Nuclear Factor*-*κB Ligand* (*RANKL*), therefore, it is essential for bone reabsorption (Boyle et al. 2003). Some studies in humans have identified SNPs in this gene associated with bone mineral density (Yamada et al. 2003; Hsu et al. 2006). Although there are evidences of the association between the *OPG* gene and bone-related traits in humans, no association studies have been conducted in chickens to date. Hence, the aim of the current study was to identify SNPs in the chicken *OPG* gene and to test the association of the most informative SNP with skeletal structure and production traits in a paternal broiler line.

## Material and methods

### Experimental populations and phenotypic traits

In this study, 10 chickens from a paternal broiler line (TT) developed by the EMBRAPA Poultry Breeding Program were used for SNP discovery. This line has been under multi-trait selection since 1992, with emphasis to improve body weight, feed conversion, carcass and cuts yield, viability, fertility and hatchability. The association analyses were performed in an experimental broiler population, called TT Reference Population. This population was generated for genomic studies by the expansion of the paternal broiler line TT. To represent the TT line, one sire from each sire family (20) and one dam from each dam family (92) were chosen to generate the TT Reference Population. Matings were conducted to avoid relatives in a proportion of 1 male to 5 females (1:5) to produce 1600 day-old chicks from 5 hatches, half of each sex. Chicks were pedigree tagged at hatching, kept in collective pens until 35 days of age, and then housed in individual cages for feed conversion evaluation. The chickens received feed and water *ad libitum*. Commercial broiler diets containing 3150 kcal/kg ME and 21% CP (1–21 days), 3200 kcal/kg ME and 20% CP (22–35 days), and 3200 kcal/kg ME and 18.5% CP (36–41 days) were provided. The lighting schedule consisted of 24 h of light during the first day; then a reduction of one hour a day until reaching natural lighting from 2 to 20 days of age; from 21 to 35 days of age light was provided from 4:00 am to 10:00 pm, and from 35 to 42 days chickens received 24 hours light. Chick individual weights were obtained at birth, 21 and 35 days of age. At 42 days of age, 1465 chickens were slaughtered and 38 traits were measured for 4 different groups of traits: 1) performance, 2) carcass and cuts, 3) internal organs, and 4) skin. In addition, 17 bone integrity-related traits were also evaluated in the tibia and femur of approximately 590 chickens. Carcass cuts yield and percentage of bones related to body weight were calculated. A total of 85 traits were evaluated in the TT Reference Population. The complete list of traits and their descriptive statistics are presented in Additional file 1: Table S1.

This research was carried out according to the ethical guidelines of the Embrapa Swine and Poultry Ethics Committee on Animal Utilization, under the protocol number 011/2011, following the international guidelines for animal welfare.

### Gene amplification and polymorphisms identification

Genomic DNA was extracted from whole blood samples using DNAzol (Invitrogen, San Diego, CA) and the DNA stock solution was stored at -20°C. DNA quality was evaluated using a 1.0% (w/v) agarose gel electrophoresis. Ten set of primers were designed based on the chicken OPG gene sequence (GenBank accession # NC_006089.3) with the goal of finding one informative SNP for genotyping, with no specific target region in the gene. When the primers were designed, their quality was evaluated *in silico* for secondary structures formation (primer-dimers, hairpins, etc.). From those, 4 pairs of primers were synthetized, and the one with the most specific and strongest band was chosen for SNP identification: primers forward (5′-TGTTTGAAGCTACCTCCTCCTGCT-3′) and reverse (5′-TCGTGCACTCCTGCTTGATGTACT-3′). A PCR reaction containing a final volume of 25 μL, with 1X reaction buffer, 2.0 mM of MgCl_2_, 0.4 mM of dNTPs, 0.2 μM of each primer, 0.06 U of Taq DNA polymerase (Invitrogen, San Diego, CA) and 30.0 ng of genomic DNA was prepared. The PCR was performed under the following conditions: denaturing at 95°C for 5 min, followed by 35 cycles of denaturing at 95°C for 1 min, annealing at 59°C for 1 min, extension at 72°C for 1 min and final extension for 10 min at 72°C, resulting in an amplicon of 704 bp (Additional file 1: Figure S1). Sequence reaction was prepared using the BigDye® Terminator v3.1 Cycle Sequencing Kit (Applied Biosystems, Foster City, CA) using forward and reverse primers. Sequencing was performed in ABI 3130xl Genetic Analyzer (Applied Biosystems, Foster City, CA). Genomic sequences from 10 TT chickens were compared using Phred/Phrap/Consed/Polyphred programs for polymorphism identification (Ewing et al. 1998; Gordon et al. 1998). Only mutations in regions with Phred quality ≥20 were considered. Alignments were done based on the chicken genome assembly ICGSC Gallus_gallus-4.0, 2011.

### Genotyping

One prospected polymorphism was chosen for genotyping based on its informativeness in the 10 parental chickens that were sequenced. Besides that, the presence of restriction enzyme sites recognizing the SNP, as well as, the number of bands and its pattern were considered to design an accurate PCR-RFLP assay. Restriction analysis was performed using NEBcutter software (Vincze et al. 2003). The PCR-RFLP assay was carried out using the *Bsr* I restriction endonuclease (New England Biolabs Inc. Beverly, MA). Digestion reaction was conducted with final volume of 25 μL, containing: 13 μL of the PCR product; 1U of the *Bsr* I and 2.5 μL of the buffer. The mixture was incubated at 65°C for 3.5 hours, following an inactivation cycle of 80°C for 20 min. After digestion, samples were analyzed in 2% (w/v) agarose gel using the 100 bp DNA ladder (Promega Corporation, Madison, WI, EUA). The parental generation (n = 112) and 1465 offspring from the TT Reference Population were genotyped for the selected SNP.

### Statistical analysis

Descriptive statistics, frequency analyses and ANOVA (to test the fixed effects), were performed in SAS 8.0 (SAS Institute, Cary, NC, EUA). Hardy-Weinberg equilibrium (HWE) was tested by comparing the expected and observed genotypic frequencies using a Chi-square test. Association analyses between the SNP g.9144C > G and the 85 evaluated traits were carried out with QxPaK v.4.0 (Perez-Enciso and Misztal 2004) which employs a maximum likelihood approach. Data were analyzed with a linear mixed model including the fixed effects of sex, hatch and SNP, and the infinitesimal and residual error as random effects. The additive (a), additive + dominant (ad) and dominant (d) effects of the SNP were tested including their interaction with sex. Association analyses were performed considering 2 and 3 genotypes for this SNP. Heritabilities and genetic correlations were estimated by the Restricted Maximum Likelihood method under a multi-trait animal model with the QxPaK program using the same mixed model described previously.

### Gene interaction network

The GeneMANIA (http://www.genemania.org) software was used to investigate the association of the *OPG* with other genes possibly related to its functions. The *OPG*, *COL1A2*, *COL14A1*, *FABP4*, *CALB1*, *NPY*, *CCK*, *ADIPOQ*, *ADIPOR1* and *ADIPOR2* were the input genes submitted to the program. These genes were selected based on their function (bone and/or fat metabolism) and physical proximity to the *OPG* as described in the literature. As the software currently does not support *Gallus gallus* information, the organism selected was *Homo sapiens*. All the datasets were collected from publicly available databases. The network weighting method was selected automatically by the software, which uses the query genes to determine which source of annotations is the most appropriate. With an input gene list of 5 or more genes, GeneMANIA assigns weights using linear regression to maximize connectivity between all query genes. The 20 genes most related to the query genes were selected to display in the network.

## Results and discussion

### SNP discovery

Eight SNPs were identified in the sequenced fragment of the *OPG* gene (Table 1) in 9 animals. The SNP information was submitted to the dbSNP in the NCBI (https://www.ncbi.nlm.nih.gov/SNP). Two SNPs were in exomic regions. Polymorphisms g.8842C > T and g.8795A > G are missense mutations. The SNP g.8842C > T alters codon TTC to TCC and the amino acid phenylamine is changed to serine. The SNP g.8795A > G changes the codon GGT to AGT and glycine is produced instead of serine. Most of the identified SNPs were firstly described in this study, except for the SNP located at 141,826,909: A > C (ss538263115).

**Table 1:** **SNPs identified in the** ***OPG*** **gene fragment**

Position (bp) ^1^	MAF ^2^	Submitted SNP (ss#)	SNP in contig	Gene region
135,912,350: A > G	A (0.21)	749616244	g.9244A > G	Intron 2
135,912,303: C > G	C (0.28)	749616245	g.9144C > G	Intron 2
135,912,295: A > T	A (0.25)	749616246	g.9051A > T	Intron 2
135,912,174: C > T	T (0.25)	749616247	g.9025C > T	Intron 2
135,912,120: A > G	A (0.29)	749616248	g.8971A > g	Intron 2
135,912,094: A > C	A (0.25)	749616249 (*ss538263115*)	g.8850A > C	Intron 2
135,912,001: C > T	T (0.19)	749616250	g.8842C > T	Exon 3
135,911,901: A > G	A (0.44)	749616251	g.8795A > G	Exon 3

^1^ICGSC Gallus_gallus-4.0, 2011.

^2^Minor Allele Frequency in 9 TT chickens.

### Genotyping, frequencies and descriptive analysis

The polymorphism located at 135,912,303 bp of the chromosome (*OPG* SNP g.9144C > G) was chosen to be genotyped in the whole TT Reference Population for association analyses. For this SNP, genotyping was carried out with the enzymatic digestion using the *Bsr I* restriction endonuclease, which generated 4 common fragments plus either two fragments of 321 and 76 bp for the G allele, or one fragment of 397 bp for the C allele. Out of 112 genotyped parental chickens, 93 animals had a well-defined genotype and therefore were used in this study, distributed as 37 GG, 50 CG and 6 CC. The allelic frequency in the parental population was G = 0.67 and C = 0.33. A total of 1230 animals out of 1465 chickens from the TT Reference Population had its genotypes obtained. The PCR-RFLP revealed the presence of 496 chickens with the homozygous genotype (GG =0.40), 665 with the heterozygous genotype (GC =0.54) and 69 were homozygous (CC =0.06). The frequency for the G and C alleles was 0.67 and 0.33, respectively. The marker studied have failed the HWE hypothesis in both generations, with p < 0.05 and p < 0.0003, respectively, for the parental and its offspring. Besides the low frequency of the CC genotype in the parental generation, no male, which contributes with more offspring to the next generation, was observed with the CC genotype in the parentals. This could explain the reduced number of offspring with the CC genotype, causing the deviation from HWE found in our study.

The TT population, even being a highly selected broiler line, contains sufficient genetic variability for gene discovery. Phenotypic variation for the 85 traits evaluated in this study (Additional file 1: Table S1), indicates the presence of a considerable amount of genetic variation among animals within this population.

### Association analyses

The models with the additive effect and the additive effect within sex had the best fit to the data. Due to the limited number of observations for the CC genotype (6%) and its unbalanced frequency distribution within dam families, the association analyses were performed considering the two most frequent *OPG* genotypes (GG and GC) and all three genotypes (GG, GC and CC). Better estimates (small standard errors) were obtained when excluding the CC genotype. Therefore, only the most frequent genotypes were included in the final analysis to avoid statistical bias.

The *OPG* g.9144C > G SNP was associated with tibia weight and tibia breaking strength, with an increase of 0.30 g and 1.42 Kg, respectively, for each C allele added to the genotype (Table 2). Also, associations of the *OPG* with abdominal fat weight and abdominal fat yield were identified with an increase of 1.9 g and 0.07%, respectively. However, the presence of the C allele had a negative effect on heart yield and drumstick muscle yield (Table 2).

**Table 2:** **Results from the significant associations of the g.9144C** > **G SNP with chicken traits evaluated in the TT Reference Population under the additive model**

Trait	n	Mean ± SD	P-value	a ± SE
AFW (g)	1150	47.80 ± 14.01	0.02	1.91 ± 0.83
AFY (%)	1149	2.15 ± 0.62	0.03	0.07 ± 0.03
GY (%)	1134	1.45 ± 0.25	0.01	0.03 ± 0.01
HY (%)	1134	0.56 ± 0.07	1.0 × 10^-3^	-0.01 ± 0.00
DMY (%)	1140	5.97 ± 0.46	0.02	-0.06 ± 0.03
TW (g)	563	11.74 ± 2.09	0.01	0.30 ± 0.12
TBS (Kg)	563	32.26 ± 7.88	0.02	1.42 ± 0.61

a = additive effect, SE = standard error.

AFW: Abdominal fat weight; AFY: Abdominal fat yield; GY: Gizzard yield; HY: Heart yield; DMY: Drumstick muscle yield; TW: Tibia weight, and TBS: Tibia breaking strength.

When the additive model within sex was fitted, positive associations were observed for body weight at 21 days in females and gizzard weight in males (Table 3). Nevertheless, the C allele had a negative effect on drumstick skin weight, drumstick skin yield, thigh muscle yield, leg yield and leg muscle yield. This effect was only identified in females. Association of *OPG* with body weight became significant only at 21 days of age. This could be due to the fact that bone length and width as well as mineral deposition reach their maximum development in the first two to three weeks of life (Angel 2007).

**Table 3:** **Results from the significant associations of the g.9144C** > **G SNP with chicken traits evaluated in the TT Reference Population under the additive model within sex**

Trait	n	Mean ± SD	P-value	a ± SE (Male)	a ± SE (Female)
BW21(g)	1140	642.45 ± 133.43	4.0 × 10^-3^	6.64 ± 6.63	17.24 ± 6.17
GW (g)	1135	32.27 ± 6.07	0.02	1.31 ± 0.50	0.70 ± 0.47
DSW (g)	1140	17.49 ± 4.35	0.03	0.04 ± 0.39	-0.93 ± 0.36
DSY (%)	1139	0.79 ± 0.17	0.02	-0.001 ± 0.01	-0.04 ± 0.01
TMY (%)	1143	10.40 ± 0.87	6.0 × 10^-3^	0.05 ± 0.07	-0.20 ± 0.06
LY (%)	1128	23.20 ± 1.21	1.0 × 10^-12^	0.01 ± 0.09	-0.22 ± 0.08
LMY (%)	1138	16.37 ± 1.10	6.0 × 10^-3^	-0.01 ± 0.08	-0.25 ± 0.08

a = additive effect, SE = standard error.

BW21: Body weight at 21 days, GW: Gizzard weight; DSW: Drumstick skin weight; DSY: Drumstick skin yield; TMY: Thigh muscle yield; LY: Leg yield, and LMY: Leg muscle yield.

The SNP in the *OPG* gene was associated with tibia weight and breaking strength, important skeletal components. Also, associations with performance, carcass and internal organs were found in this study. Furthermore, interactions between the SNP and sex using the additive model for some of the traits were identified (Table 3). These results are in agreement with human studies, where an interaction between the *OPG* and sex was also observed (Beyens et al. 2007; Hsu et al. 2006). Four SNPs were identified in the promoter region of *OPG*, which were associated with bone mineral density (BMD) only in men (Hsu et al. 2006). Yamada and coleagues (Yamada et al. 2003) also identified SNPs in the promoter region of the *OPG* gene, whose association with BMD was significantly only in women. Beyens and collaborators (Beyens et al. 2007) found an association of a polymorphism in the *OPG* gene with Paget’s disease of bone (PDB), which is a late-onset skeletal disorder. In that study, no association was detected in men and highly significant association was found in women.

The interaction between *OPG* and sex observed in our study might be explained by the large influence of the female sex hormone estrogen in the *OPG* expression. Estrogen stimulates the osteoblastic human cells to increase osteoprotegerin secretion (Hofbauer et al. 1999). Besides the reproductive functions, estrogen also has an important role on female skeleton (Khosla 2010; Turner et al. 1994). Although there is low production of this hormone in males, estrogen also affects the male skeletal structure (Khosla et al. 2002).

Associations with fat deposition-related traits, such as abdominal fat, drumstick and thigh skin weights and their yields were also identified (Tables 2 and 3). The main sites for fat deposition in chickens are the abdomen and the subcutaneous tissue. According to Tumova and Teimouri ( 2010), abdominal fat is 20% of total body fat in broilers and subcutaneous fat is 18%. In our population we did not measure the subcutaneous fat. However, the skin weight of several cuts was evaluated. To verify the relationship between abdominal fat and skin weights, we estimated the genetic correlations and heritability for those traits (Table 4). Our results indicate the existence of a positive moderate genetic correlation between AFW and skins (DSW and TSW), of 0.28 and 0.40, respectively (Table 4). These findings are in agreement with those of Zerehdaran et al. ( 2004) who observed high genetic correlation between abdominal fat weight and skin weight (0.54), indicating that some of the genes controlling abdominal fat weight also regulate skin fat weight.

**Table 4:** **Heritability estimates (diagonal) and genetic correlations (above the diagonal) for body weight, abdominal fat weight, and drumstick and thigh skin weights**

Traits	BW42	AFW	DSW	TSW
**BW42**	0.35	0.38	0.53	0.62
**AFW**		0.33	0.28	0.40
**DSW**			0.17	0.44
**TSW**				0.28

BW42 – body weight at 42 days of age, AFW – abdominal fat weight, DSW – drumstick skin weight, TSW – thigh skin weight.

Heritabilities were moderate for AFW and TSW, and low for DSW (Table 4), indicating the existence of enough genetic variability to allow for genetic progress by selection against fat deposition. The moderate genetic correlations between BW42 and fat weight and skin weights indicate that selection that have been applied for increased BW42 are greatly increasing fat accumulation in broilers.

### Understanding the mechanisms of associations

The osteoprotegerin gene (*TNFRSF11B*) is located on chicken chromosome 2 (GGA2) at 135,904,344- 135,921,143 bp (NC_006089.3), spanning 20 kb (*Gallus gallus*, assembly ICGSC Gallus_gallus-4.0 (GCA_000002315.2)). In this region, several QTL have already been identified for traits associated with bone structure in chickens, such as tibia mineral density (Zhou et al. 2007), mineral content and tibia width (Schreiweis et al. 2005), and tibia length (Zhou et al. 2007). Other QTL not directly related to bone structure were also mapped within this region: weight gain (Ou et al. 2009), breast muscle weight (Tercic et al. 2009) and gizzard weight (Navarro et al. 2005; Pinto et al. 2006), being the last one confirmed in our study.

The *OPG* gene is an important modulator of chicken bone metabolism and there are evidences of its association with bone-related traits in humans. It is possible that the g.9144C > G SNP might be directly involved with the phenotypic variation observed in the present study. Despite the fact that the analyzed SNP is not located in an exomic region of the *OPG*, it could affect the expression of this gene since even a non-expressed gene region can interfere in gene expression.

Moreover, as a TNF family member, the *OPG* gene has been associated with different biological processes besides those related to bone metabolism. The protein encoded by this gene is expressed in many tissues besides the osteoblasts, such as: heart, kidney, liver, spleen, and bone narrow (Wada et al. 2006). This fact reinforces the putative importance of the *OPG* in biological processes of different tissues other than bone.

Another possibility is that the SNP associated with those traits is in linkage disequilibrium (LD) with another polymorphism, which could be the causative mutation for the observed phenotypes. To support this hypothesis, important genes involved with bone formation and remodeling are located near the *OPG* in GGA2, as *collagen alpha*-*2*(*I*) *chain* (*COL1A2*), *collagen alpha*-*1* (*XIV*) *chain* (*COL14A1*) and *Calbindin* (*CAL*). Moreover, in GGA2, important genes involved with fat metabolism and energy homeostasis were mapped: *fatty acid binding protein 4* (*FABP4*), *neuropeptide Y* (*NPY*) *and cholecystokinin* (*CCK*), which could be responsible for the associations related to fatness found in our study.

Despite of this, association with abdominal fat could be an insight about the possible interaction between adipose and bone tissues. The *adiponectin* (*ADIPOQ*), an important member of the adipocytokine family, has emerged as a key element in the regulation of bone metabolism, although its functional mechanism has not been completely elucidated. According to Luo et al. ( 2006), in humans, ADIPOQ stimulates bone reabsorption by two mechanisms: 1) the adiponectin hormone inhibits the *OPG* gene expression in osteoblasts and 2) stimulates the *RANKL* that is an osteoclast differentiation factor.

Another important hormone expressed in adipocytes that is supposed to have effect on bone metabolism is Leptin (Lamghari et al. 2006). Despite the *leptin* (*LEP*) gene has not yet been successfully mapped in the chicken genome and all the controversy over the avian *LEP* gene that still exists (Scanes 2008), a leptin-like protein has been described in chickens (Neglia et al. 2008). In a review about Leptin, Hamrick and Ferrari ( 2008) concluded that Leptin and the sympathetic system have dual effects on the skeleton and provides an important mechanism that links bone metabolism and body composition. Lamghari et al. ( 2006) suggested that *LEP* gene modulates positively the *OPG*/*RANKL* balance by inhibiting the expression of *RANKL* gene. In humans, there is an inverse relationship between bone mass and fat mass (Zhao et al. 2007). The hypothesis that excessive lipid consumption affects bone metabolism in laying hens has been tested (Jiang et al. 2013) and results indicated that a high energy and low protein diet induced a fatty liver disorder with an up regulation in bone turnover and exacerbated skeletal damage.

**Figure 1:**
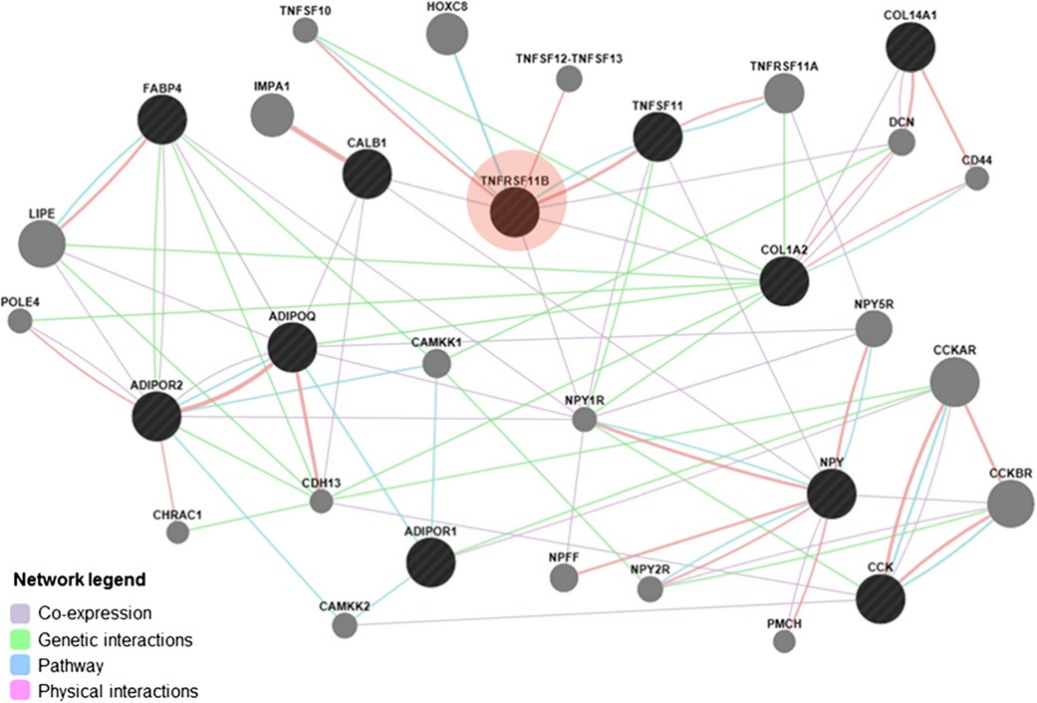
***OPG*** **(** ***TNFRSF11B*** **; in red) gene network.** Circles represent genes and connecting lines represent interactions between genes. Black circles represent the set of genes provided to the GeneMania software. Gray circles are the extra genes added to the network by the program that are strongly connected to query genes.

Production traits are usually controlled by multiple genes in the genome. To improve the discussion about the possible relationship of the *OPG* with the set of genes discussed above, a gene network was performed with GeneMania (http://www.genemania.org; Zuberi et al. 2013). The network which was constructed considering gene interactions, co-expression and pathways is shown in Figure 1.

This network shows that effectively the genes discussed previously are related to the OPG. In addition to that, other genes appeared enriching the network, which can be helpful to explain the association results obtained. For instance, *decorin* (*DCN*), *calcium*/*calmodulin*-*dependent protein kinase kinase 1 alpha* (*CAMKK1*) and *neuropeptide Y receptor Y1* (*NYP1R*) genes are all related to the *OPG*. As seen in the network, *COL1A2*, *COL14A1* and *CALB1* co-express with *OPG*, showing that, besides the possibility of LD, the association with traits might also be due to the interaction of these genes with the *OPG*. The same occurs with the fat-related genes *FABP4*, *NPY* and *CCK*. Besides being located on GGA2, these genes are also involved in the *OPG* network, interacting with *ADIPOQ*, *ADIPOR1* and *ADIPOR2*, which reinforces the associations found in our study with fat-related traits. In our study, the network constructed clearly highlights the relevance of the gene studied and shows other genes interacting with the *OPG*, which provides useful information about the mechanisms of action of genes that can be used in future research.

Finally, the associations of the *OPG* SNP with several traits identified in this study might be due to a direct effect caused by a mutation in the *OPG* gene, interactions with other genes as shown in the network or an indirect effect caused by LD between the *OPG* SNP and the causative mutation. Further studies are needed to elucidate the possible causes of the associations found. This SNP could be considered as a potential genetic marker to improve tibia weight and resistance. However, caution should be taken because of its negative effect in other important traits in poultry, such as abdominal fat and leg muscle development, especially in females. Validation of these findings in future generations or in an unrelated population could clarify if the contrasting effects of this SNP are due to pleiotropy or linkage. Moreover, the consistent associations observed in a pure line, together with its location in a QTL region for bone integrity in chickens and its important biological function, indicate that this gene might be directly responsible for the significant associations found, at least for the bone-related traits as tibia weight and breaking strength. Altogether, these results suggest that the *OPG* SNP has a significant effect on skeletal structure and other important traits in broilers. Furthermore, these results suggest a possible involvement of the *OPG* gene in fat deposition in poultry.

## Electronic supplementary material

Additional file 1: Table S1: Descriptive statistics of production traits evaluated in the TT broiler Reference Population. **Figure S1.** Amplicon size with primers to amplify the *OPG* gene. (DOCX 35 KB)
