# Supplementary material for: Unraveling the associations of osteoprotegerin gene with production traits in a paternal broiler line
Source: Springerplus. 2014 Nov 20;3:682. doi: 10.1186/2193-1801-3-682 (PMC4247828; doi:10.1186/2193-1801-3-682)
Supplement: Supplementary file 1 — Additional file 1: Table S1: Descriptive statistics of production traits evaluated in the TT broiler Reference Population. Figure S1. Amplicon size with primers to amplify the OPG gene. (DOCX 35 KB) [file 40064_2014_1374_MOESM1_ESM.docx]

**Supplementary material**

**Table 1** Descriptive statistics of production traits evaluated in the TT broiler Reference Population

| **Traits** | **N** | **Mean** | **SD** | **Min** | **Max** |
| --- | --- | --- | --- | --- | --- |
| **Performance** | | | | | |
| Birth Weight (g) | 1156 | 47.7 | 3.7 | 37.4 | 61.8 |
| Body weight at 21 days (g) | 1140 | 642.4 | 133.4 | 326.0 | 998.0 |
| Body weight at 35 days (g) | 1158 | 1727.9 | 200.1 | 922.0 | 2300.0 |
| Body weight at 41 days (g) | 1153 | 2218.4 | 248.5 | 1122.0 | 2908.0 |
| Weight at 42 days (g) | 1160 | 2224.8 | 257.2 | 1102.0 | 2919.0 |
| Feed consumption (35-41 days) (g) | 1153 | 1094.7 | 147.5 | 508.0 | 1590.0 |
| Weight gain (35-41 days) (g) | 1149 | 490.7 | 103.7 | 158.0 | 802.0 |
| Feed conversion ratio (35-41 days) | 1149 | 2.3 | 0.5 | 1.4 | 5.0 |
| **Carcass and cuts** | | | | | |
| Weight after bleeding and plucking (g) | 1154 | 2056.8 | 244.6 | 1020.0 | 2711.0 |
| Weight of blood and feathers (g) | 1150 | 168.7 | 26.3 | 82.0 | 293.0 |
| Carcass weight (g) | 1149 | 1640.4 | 199.1 | 762.1 | 2212.0 |
| Abdominal fat weight (g) | 1150 | 47.8 | 14.0 | 8.0 | 94.0 |
| Head weight (g) | 1139 | 52.6 | 7.3 | 35.6 | 77.2 |
| Feet weight (g) | 1137 | 74.5 | 13.7 | 36.3 | 108.1 |
| Drumette weight (g) | 1145 | 85.6 | 10.9 | 50.2 | 116.6 |
| Mid-joint-wing weight (g) | 1138 | 61.9 | 7.9 | 30.8 | 87.8 |
| Wing tip weight (g) | 1145 | 19.8 | 2.9 | 10.2 | 31.8 |
| Cooled tibia weight (g) | 1145 | 55.7 | 10.2 | 27.8 | 85.4 |
| Drumstick muscle weight (g) | 1141 | 133.0 | 20.5 | 52.8 | 208.0 |
| Cooled femur weight (g) | 1146 | 32.4 | 5.6 | 18.0 | 56.6 |
| Thigh muscle weight (g) | 1144 | 232.1 | 37.7 | 92.6 | 351.2 |
| Breast muscle weight (g) | 1043 | 294.1 | 42.2 | 142.7 | 428.6 |
| Inner fillets weight (g) | 1147 | 77.3 | 11.8 | 38.4 | 114.9 |
| Breast bone weight (g) | 1147 | 98.1 | 15.0 | 47.3 | 152.7 |
| Back weight (g) | 1140 | 263.4 | 35.3 | 135.6 | 370.7 |
| Neck weight (g) | 1145 | 119.9 | 21.0 | 46.0 | 200.4 |
| Leg weight (g) | 1129 | 516.8 | 73.3 | 232.6 | 718.0 |
| Leg muscle weight (g) | 1139 | 365.0 | 55.5 | 157.6 | 529.4 |
| Drumstick weight (g) | 1135 | 206.0 | 30.9 | 86.2 | 306.6 |
| Thigh weight (g) | 1140 | 310.7 | 45.8 | 130.8 | 462.8 |
| Breast weight (g) | 1140 | 501.1 | 62.7 | 253.7 | 710.8 |
| Leg yield (%) | 1128 | 23.2 | 1.2 | 18.4 | 27.6 |
| Leg muscle yield (%) | 1138 | 16.4 | 1.1 | 12.2 | 19.9 |
| Blood and feather percentage (%) | 1150 | 7.6 | 1.0 | 3.9 | 11.3 |
| Carcass yield (%) | 1149 | 73.7 | 1.6 | 63.6 | 79.8 |
| Abdominal fat yield (%) | 1149 | 2.2 | 0.6 | 0.5 | 4.7 |
| Head percentage (%) | 1138 | 2.4 | 0.2 | 1.7 | 3.3 |
| Feet percentage (%) | 1136 | 3.3 | 0.4 | 2.0 | 4.4 |
| Drumette yield (%) | 1144 | 3.9 | 0.3 | 2.6 | 5.5 |
| Mid-joint-wing yield (%) | 1137 | 2.8 | 0.2 | 2.0 | 3.5 |
| Wing weight (%) | 1136 | 167.2 | 19.5 | 91.6 | 223.8 |
| **Traits** | **N** | **Mean** | **SD** | **Min** | **Max** |
| Wing yield (%) | 1135 | 7.5 | 0.4 | 6.0 | 9.2 |
| Cooled tibia yield (%) | 1144 | 2.5 | 0.3 | 1.7 | 3.9 |
| Drumstick muscle yield (%) | 1140 | 6.0 | 0.5 | 4.5 | 8.7 |
| Drumstick yield (%) | 1134 | 9.3 | 0.6 | 7.3 | 12.1 |
| Cooled femur yield (%) | 1145 | 1.5 | 0.2 | 1.0 | 2.3 |
| Thigh muscle yield (%) | 1143 | 10.4 | 0.9 | 5.6 | 13.1 |
| Thigh yield (%) | 1139 | 13.9 | 1.0 | 9.7 | 16.9 |
| Breast muscle yield (%) | 1142 | 13.2 | 1.2 | 9.2 | 17.5 |
| Inner fillets yield (%) | 1146 | 3.5 | 0.4 | 2.1 | 5.2 |
| Breast bone yield (%) | 1146 | 4.4 | 0.5 | 2.7 | 6.3 |
| Breast yield (%) | 1138 | 22.5 | 1.4 | 17.3 | 27.1 |
| Back percentage (%) | 1139 | 11.8 | 0.9 | 8.0 | 15.0 |
| Neck percentage (%) | 1144 | 5.4 | 0.7 | 3.2 | 7.8 |
| **Skin** | | | | | |
| Drumstick skin weight (g) | 1140 | 17.5 | 4.4 | 5.6 | 36.6 |
| Thigh skin weight (g) | 1142 | 46.3 | 9.6 | 14.2 | 78.8 |
| Breast skin weight (g) | 1144 | 31.7 | 6.7 | 10.3 | 61.7 |
| Drumstick skin yield (%) | 1139 | 0.8 | 0.2 | 0.3 | 1.5 |
| Thigh skin yield (%) | 1141 | 2.1 | 0.4 | 0.9 | 3.5 |
| Breast skin yield (%) | 1143 | 1.4 | 0.2 | 0.6 | 2.4 |
| **Internal Organs** | | | | | |
| Lungs weight (g) | 1143 | 15.3 | 3.0 | 6.6 | 24.6 |
| Liver weight (g) | 1136 | 52.4 | 8.7 | 25.4 | 82.4 |
| Heart weight (g) | 1135 | 12.4 | 2.1 | 7.4 | 19.2 |
| Gizzard weight (g) | 1135 | 32.2 | 6.1 | 17.8 | 56.1 |
| Liver yield (%) | 1135 | 2.4 | 0.3 | 1.3 | 3.3 |
| Heart yield (%) | 1134 | 0.6 | 0.1 | 0.4 | 0.9 |
| Lungs yield (%) | 1142 | 0.7 | 0.1 | 0.4 | 1.1 |
| Gizzard yield (%) | 1134 | 1.5 | 0.3 | 0.9 | 2.4 |
| **Bone Integrity** | | | | | |
| Tibia dry weight (g) | 563 | 11.7 | 2.1 | 7.3 | 17.1 |
| Tibia length (mm) | 563 | 95.3 | 3.9 | 80.0 | 106.2 |
| Tibia width (mm) | 563 | 8.7 | 0.9 | 6.0 | 11.6 |
| Tibia breaking strength (kg) | 563 | 32.3 | 7.9 | 16.3 | 58.5 |
| Tibia breaking area (mm^2^) | 563 | 68.3 | 23.9 | 20.2 | 163.3 |
| Tibia breaking distance (mm) | 563 | 3.64 | 1.16 | 1.80 | 15.06 |
| Tibia dry matter content (%) | 562 | 50.5 | 3.3 | 39.5 | 64.0 |
| Tibia ash content (%) | 563 | 22.2 | 1.5 | 17.6 | 28.0 |
| Dry femur weight (g) | 562 | 8.5 | 1.4 | 4.4 | 12.7 |
| Femur length (mm) | 562 | 69.5 | 3.1 | 59.3 | 78.0 |
| Femur width (mm) | 563 | 8.9 | 0.7 | 7.0 | 11.4 |
| Curvature femur score | 563 | 1.1 | 0.3 | 1.0 | 2.0 |
| Femur breaking strength (kg) | 562 | 28.7 | 5.8 | 13.4 | 52.7 |
| Femur breaking area (mm^2^) | 562 | 42.5 | 17.2 | 11.2 | 135.7 |
| Femur breaking distance (mm) | 562 | 2.96 | 1.10 | 1.51 | 15.74 |
| Femur dry matter content (%) | 563 | 51.8 | 3.5 | 43.3 | 61.7 |
| Femur ash content (%) | 563 | 21.38 | 1.56 | 15.3 | 28.60 |

**Figure 1.** Amplicon size with primers to amplify the *OPG* gene

***TGTTTGAAGCTACCTCCTCCTGCT***GATGGGGACTGCCATGTTGTGCCCCCAGAGCTGCCACATTTCAGTTCAAGATTAAATGGAAGGCAATAAAAGTCACTTATATTATTCTAGCCAGGTACCATTACTGCTACTGGTACAGTATGTGTCAGATTTGTTTGCTCAGGTACTTGCTATTTCTTTTGTGTTTGAAGTGATTGATACTCAAGAACTCCAGTGATATTAAAAGGGTATAAGTGGGTGAAGAATGGCTCAGTAAGATCCTAAATTTTTCCCATTATGAAACAGATGTGGGAGAGACCAGAAGCCGGTCTAACAGAAACATATTTTTGAAGATAGTGACATGGTTAGGCTCATAAACTCATCAAAATGTAGGTTTGTAAGTGACTGTGGTGCTCACTGAACTGAAGCAAGCAATGTTTTCTGTTTCCGTGGCTAGCTCTTGGACATCTCAGTCAAGTGGAGCATCCAGGACATCTCTCCCCCCAAGTATCTCCATTACGACCCGGGGACATCTCGTCAGGTGATGTGCAACCAGTGCCCTCCTGGGAGCTACGTAAAGCAGCACTGTACGGCTGCCAGCCCAACGGTGTGTGCTCCGTGCCCAGACCAGTACTACGCTGAAGACTGGAACAGCAACGACGAGTGCCAGTACTGCAGTGCTGTTTGCAAAGAGCTGC***AGTACATCAAGCAGGAGTGCACGA***
